# Supplementary material for: Latent representations in hippocampal network model co-evolve with behavioral exploration of task structure
Source: Nat Commun. 2024 Jan 23;15:687. doi: 10.1038/s41467-024-44871-6 (PMC10806076; doi:10.1038/s41467-024-44871-6)
Supplement: Supplementary file 1 — Supplementary Information [file 41467_2024_44871_MOESM1_ESM.pdf]

Supplementary Figures

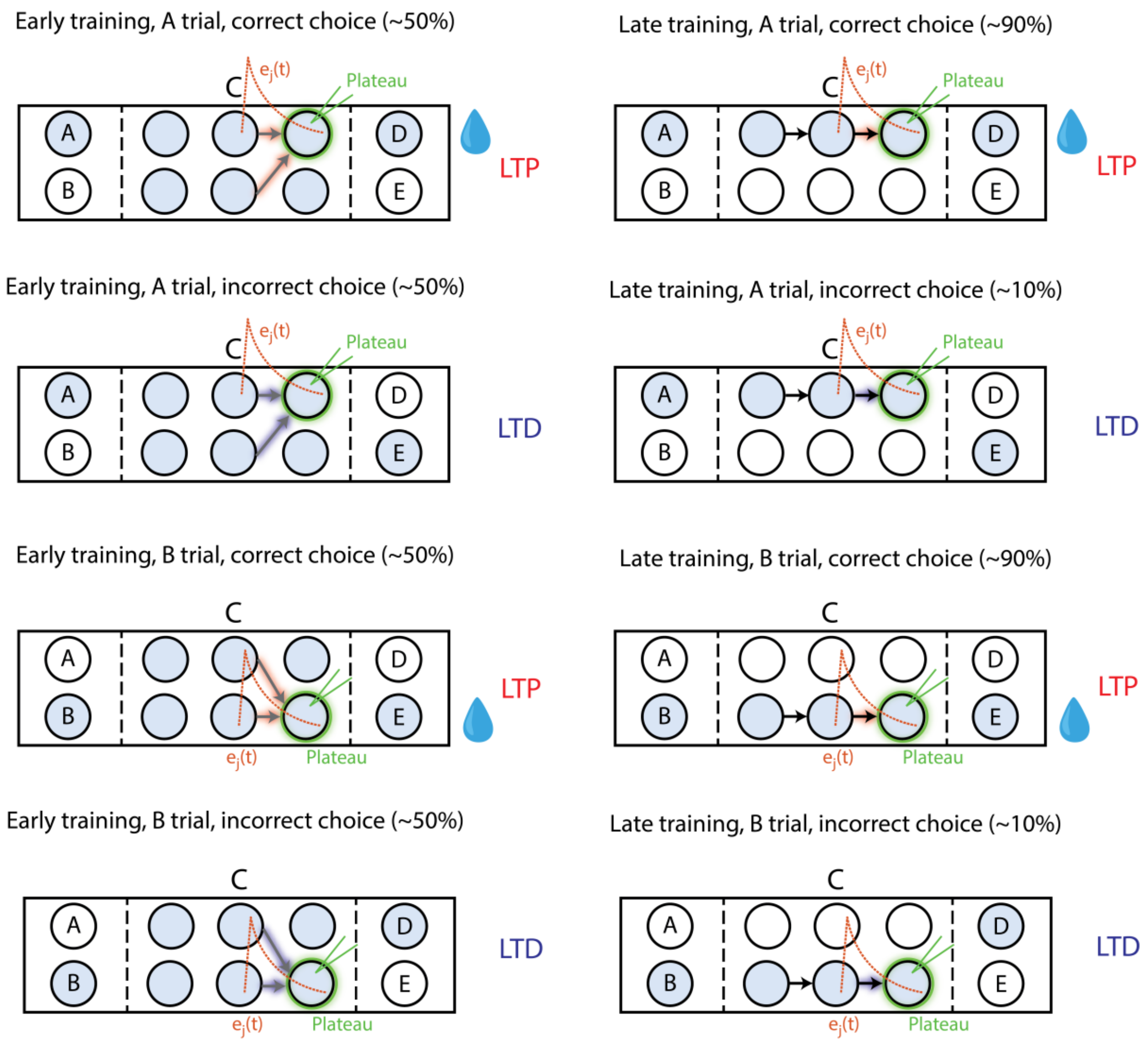

Supplementary Figure 1 – Network Learning during Early and Late Training

Network schematic for the four possible trial types and outcomes, in early training (left), and late training (right). Filled in circles represent neurons active in a given condition. A and B neurons represent the cue, while D and E neurons represent the possible reward locations. Pool of neurons representing locations in C are separated visually into potential A splitters (top row), or potential B splitters (bottom row). Plateau induction of a particular neuron(green) activates eligibility traces of previously active neurons (orange), creating potential weight updates (grey arrows). If the agent receives a reward, the weights are potentiated (red tint), whereas if the agent does not, the weights are depressed (blue tint). Early in training, the frequency of correct and incorrect choices is even, leading to no net change in weights over trials. Late in learning, splitters have formed and behavior has adjusted to the task, leading to many more correct choices than incorrect choices. As a result, the plateau acts to mostly potentiate the incoming weights onto the neuron. This reinforces the causal structure of weights. The agent breaks out of the early training phase when stochastic fluctuations in behavior accumulate as to act as a symmetry breaker to the representation, leading to the "zippering" process described in Supplementary Figure 2.

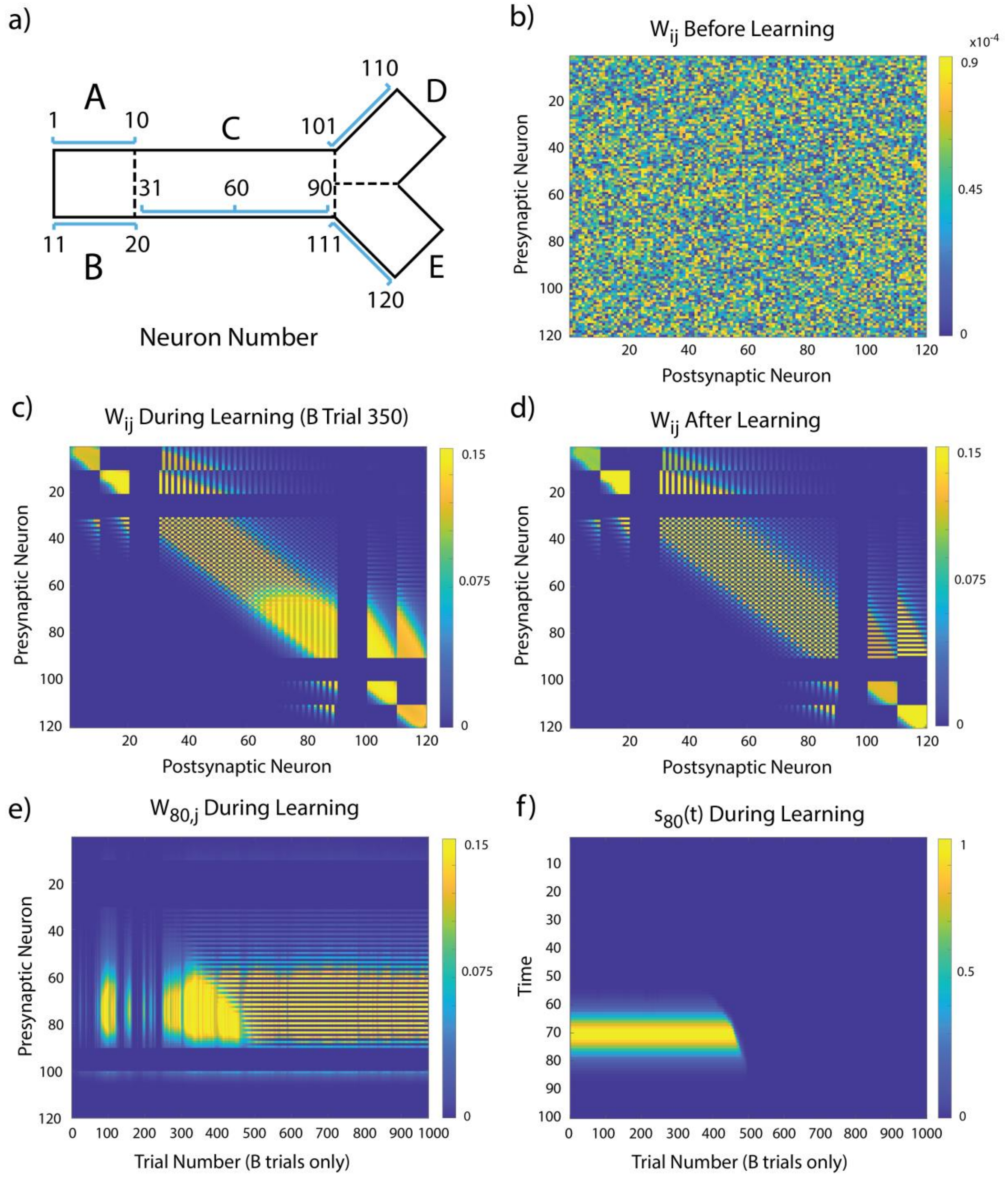

Supplementary Figure 2 – Recurrent Weights Evolve to Form Splitters

a) Neural index positions in the maze. Index is determined by the input matrix M which assigns external positions to HPC neurons. Neurons 31-90 are candidate splitters representing positions in zone C. **b)** Recurrent weights before training are small and random, meaning somatic activity is not initially context dependent. **c)** During learning, weights begin to "zipper" as the A- and B- related sequences split from each other. For this trial (trial 350 of B), the early neurons in the sequence have already split, but one close to the end of the track are still acting as generic place fields. **d)** After learning, all the neurons that represent locations within the track have split and receive inputs from other cells that share their cue identity. **e)** Evolution of weights that synapse onto neuron 80. Between trials 300 and 500, neuron 80 begins to selectively receive inputs from previous splitter neurons

which are sensitive to the same cue identity (A). **f)** As the weights evolve, the somatic activity of neuron 80 begins to disappear from B trials, before becoming a complete splitter around trial 500.

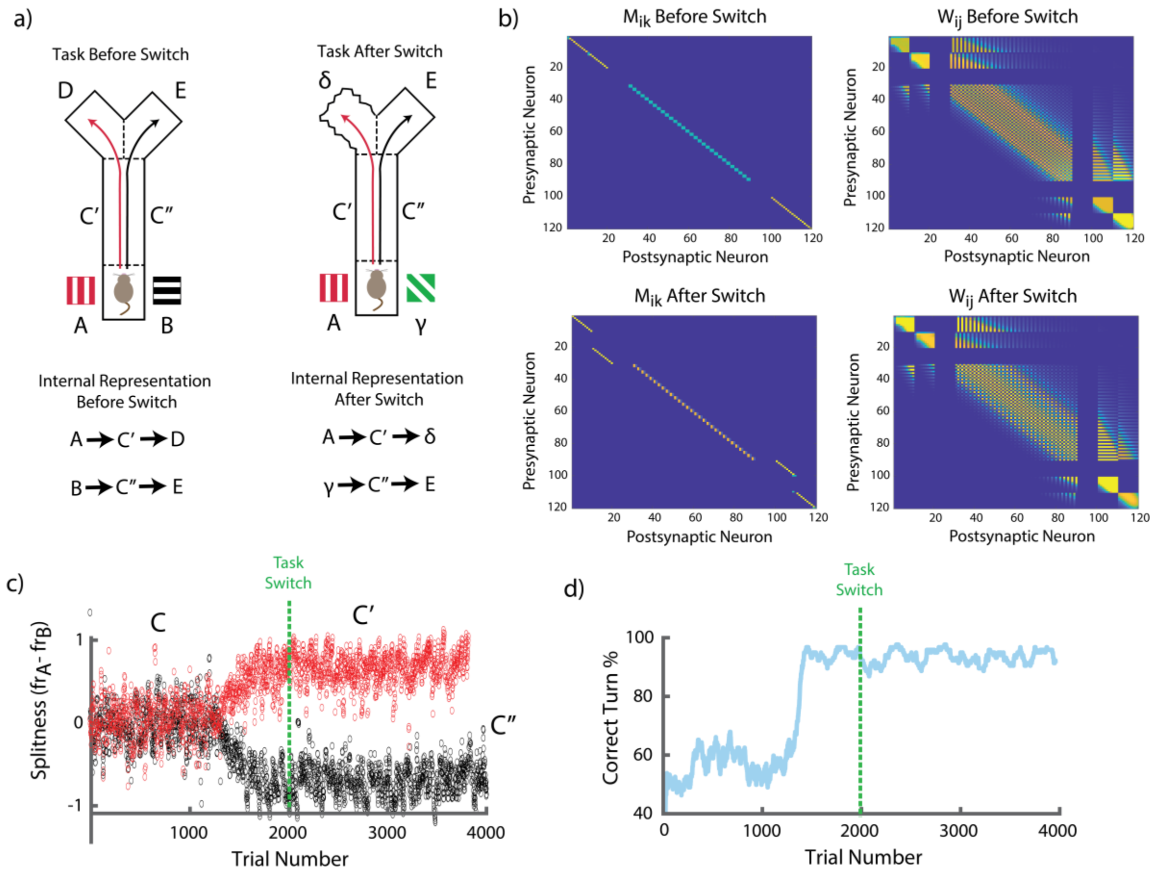

Supplementary Figure 3 – Network Generalizes to Novel External Inputs Which Match the Task Structure

a) Top, schematic of the task before and after novel external inputs (gamma and delta) are introduced. Bottom, since the new inputs do not change the structure of the task, the internal representation of the task should not change after the switch. **b)** Input weights and recurrent weights, before and after the switch. The input weights that synapse onto neurons 11-20 and 101-110 learn to be responsive to a different population of presynaptic input neurons (the population representing the new cues). The recurrent weights are largely unchanged before and after the introduction of the novel cues. **c)** Evolution of splitness over the course of training. Notably, input learning increases the variance in the neural activity from trial to trial. This is perhaps unsurprising, as now input learning, recurrent learning, and behavioral learning are simultaneously interacting with each other. Nevertheless, the splitness of the representation is maintained after the switch to the novel cues. **d)** Evolution of behavior over the course of training. Behavior is unaffected by the switch to novel cues.

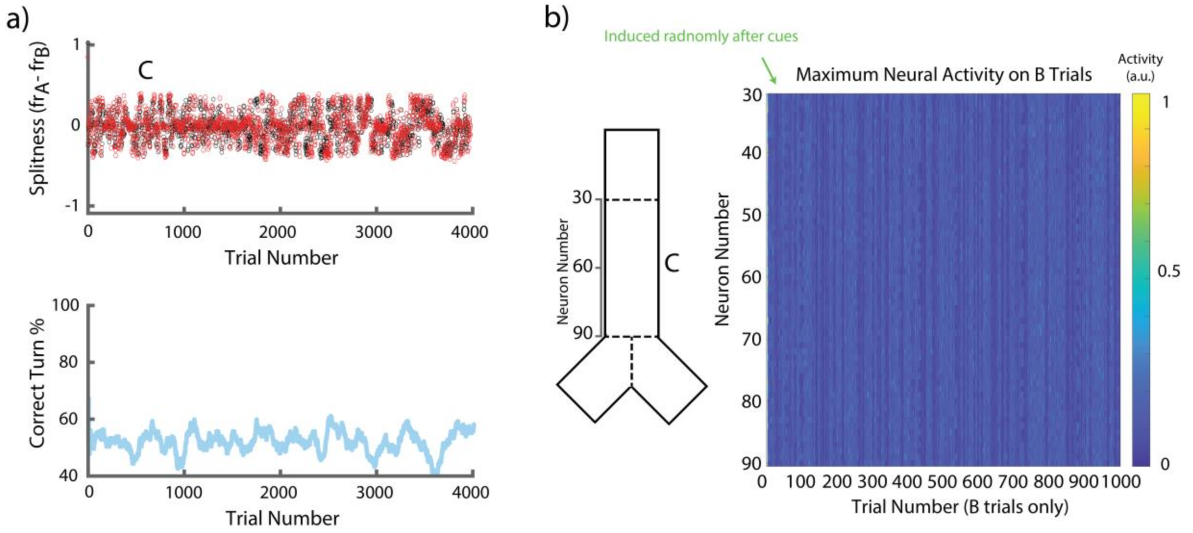

Supplementary Figure 4 – Random Induction Protocol is Insufficient to Form Splitters

a) Top, difference in firing rates (activity) on A and B cue trials ("splitness") of the two populations over the course of learning. Bottom, behavioral performance, shown as the percentage of correct turns over the course of learning. Agent fails to maximize reward in the task over 4000 trials, with a random plasticity protocol. **b)** Left inset, neuron index corresponding to a given location in the track. Right, evolution of population activity on B trials over the course of training. Plateau events are induced randomly amongst the neural population (random in both trial identity and location), and the resulting representation fails to split.
